# Evaluation of a Culture-Dependent Algorithm and a Molecular Algorithm for Identification of Shigella spp., Escherichia coli, and Enteroinvasive E. coli

**DOI:** 10.1128/JCM.00510-18

**Published:** 2018-09-25

**Authors:** Maaike J. C. van den Beld, Richard F. de Boer, Frans A. G. Reubsaet, John W. A. Rossen, Kai Zhou, Sjoerd Kuiling, Alexander W. Friedrich, Mirjam A. M. D. Kooistra-Smid

**Affiliations:** a Infectious Disease Research, Diagnostics and laboratory Surveillance, Centre for Infectious Disease Control, National Institute for Public Health and the Environment, Bilthoven, The Netherlands; b Medical Microbiology, University of Groningen, University Medical Center Groningen, Groningen, The Netherlands; c Department of Medical Microbiology, Certe, Groningen, The Netherlands; d Collaborative Innovation Center for Diagnosis and Treatment of Infectious Diseases, State Key Laboratory for Diagnosis and Treatment of Infectious Disease, The First Affiliated Hospital, School of Medicine, Zhejiang University Hangzhou, Hangzhou, China; University Hospital Münster

**Keywords:** EIEC, Escherichia coli, Shigella, whole-genome sequencing, enteroinvasive E. coli, identification, molecular methods, phenotypic methods

## Abstract

Identification of Shigella spp., Escherichia coli, and enteroinvasive E. coli (EIEC) is challenging because of their close relatedness. Distinction is vital, as infections with Shigella spp. are under surveillance of health authorities, in contrast to EIEC infections. In this study, a culture-dependent identification algorithm and a molecular identification algorithm were evaluated. Discrepancies between the two algorithms and original identification were assessed using whole-genome sequencing (WGS). After discrepancy analysis with the molecular algorithm, 100% of the evaluated isolates were identified in concordance with the original identification. However, the resolution for certain serotypes was lower than that of previously described methods and lower than that of the culture-dependent algorithm. Although the resolution of the culture-dependent algorithm is high, 100% of noninvasive E. coli, Shigella sonnei, and Shigella dysenteriae, 93% of Shigella boydii and EIEC, and 85% of Shigella flexneri isolates were identified in concordance with the original identification. Discrepancy analysis using WGS was able to confirm one of the used algorithms in four discrepant results. However, it failed to clarify three other discrepant results, as it added yet another identification. Both proposed algorithms performed well for the identification of Shigella spp. and EIEC isolates and are applicable in low-resource settings, in contrast to previously described methods that require WGS for daily diagnostics. Evaluation of the algorithms showed that both algorithms are capable of identifying Shigella species and EIEC isolates. The molecular algorithm is more applicable in clinical diagnostics for fast and accurate screening, while the culture-dependent algorithm is more suitable for reference laboratories to identify Shigella spp. and EIEC up to the serotype level.

## Abstract (precis)

Identification of Shigella spp., Escherichia coli, and enteroinvasive E. coli (EIEC) is challenging because of their close relatedness. Distinction is vital, as infections with Shigella spp.

## INTRODUCTION

In 1898, Kiyoshi Shiga first described Shigella dysenteriae as the etiologic agent of dysentery (1). Nowadays, the genus Shigella comprises four species based on antigenic properties, Shigella dysenteriae, Shigella flexneri, Shigella boydii, and Shigella sonnei. All species cause symptoms varying from mild diarrheal episodes to dysentery (2).

The relatedness of Shigella spp. with Escherichia coli has always been recognized (3–6). In addition, in the 1940s, an E. coli pathotype was described that has the same invasive mechanism as Shigella species. This pathotype was named enteroinvasive E. coli (EIEC) and is more related to Shigella spp. than noninvasive E. coli (7). EIEC and Shigella spp. possess the same virulence genes, which are located on the chromosome and carried by a large invasion plasmid (pINV) (8).

The close relatedness of Shigella spp. and E. coli challenges identification if they are encountered in laboratories. Nowadays, an initial molecular screening of fecal samples is often used for the detection of Shigella spp., in which the *ipaH* gene is a frequently used target (9–11). This is a multicopy virulence gene present on both the chromosome and pINV of Shigella spp. and EIEC strains and not present in commensal or other pathotypes of E. coli (12). Consequently, the *ipaH* gene can distinguish Shigella spp. from all pathotypes of E. coli, except for EIEC. After this initial screening, most laboratories perform culture to select Shigella and EIEC isolates for differentiation and antibiotic resistance profiling. Species identification of a selected isolate is traditionally based on phenotypical key characteristics, including motility, lysine decarboxylase, and the ability to produce both gas and indole, which are negative for Shigella spp. and usually positive for E. coli (13, 14). Unfortunately, EIEC isolates can either be positive or negative for these features (15).

In many countries, it is obligatory to notify health authorities if a laboratory confirms a case of shigellosis. In contrast, infections with EIEC are not notifiable. Therefore, a diagnostic algorithm able to distinguish Shigella spp. from E. coli, including EIEC, is required.

In the last decade, multiple molecular identification methods for Shigella spp. and E. coli, including EIEC, were reported (5, 6, 8, 16–19). One of these methods is based on the presence of the *uidA* and *lacY* genes (16, 19). However, this method appeared to be not as accurate as expected (6). Alternatively, a few research groups used whole-genome sequencing (WGS) for the distinction of Shigella spp. from E. coli (5, 6, 17, 18). Although some methods based on WGS analysis showed effectiveness, the described identification markers are phylogenetic clade specific rather than species specific (5, 6, 8). In another study, identification markers were identified by a BLAST search of coding regions of genomes of the different species (17). Consequently, these identification markers were species specific instead of clade specific; however, they were validated using only one EIEC isolate (17). Pettengill et al. (6) used a k-mer-based approach to distinguish between Shigella spp. and E. coli; however, some EIEC isolates were incorrectly identified as Shigella spp. by this approach (18). In conclusion, differentiation of Shigella spp. and E. coli, and of Shigella and EIEC in particular, is a challenge.

Despite it being proven before that Shigella spp. and EIEC are related and that EIEC is a diverse pathotype (5, 6, 8, 18), distinction is necessary for infectious disease control measures, as in many countries, shigellosis is a notifiable disease, in contrast to infections with EIEC. In this study, a culture-dependent identification algorithm was developed, based on previously described molecular, phenotypical, and serological features of Shigella spp. and EIEC. In addition, this algorithm was compared to a recently developed molecular identification algorithm (R. F. de Boer, M. J. C. van den Beld, W. de Boer, M. C. Scholts, K. W. van Huisstede-Vlaanderen, A. Ott, and A. M. D. Kooistra-Smid, unpublished data) for the identification of Shigella spp., E. coli, and EIEC.

## MATERIALS AND METHODS

### Isolates and original identification.

The selection of isolates was based on Shigella serotype or E. coli O type and is listed in Table 1. For selection, the original identification was a guide. This original identification was established with different methods at different institutes spanning the last 50 to 60 years. Most documentation about the methods used is lost. Therefore, except for the purchased isolates, the original identification cannot be considered the gold standard, and only concordance or discordance with the results obtained by the here-described algorithms can be examined.

**TABLE 1:** Original identification and original collection of the isolates used in this study

Genus and species	Strain	Serotype^*a*^	Original collection^*b*^
S. dysenteriae	CIP 57.28^T^	1	CIP
	A1	1	CDC → Cib
	A2	2	CDC → Cib
	A3	3	CDC → Cib
	A4	4	CDC → Cib
	A5	5	CDC → Cib
	A6	6	CDC → Cib
	A7	7	CDC → Cib
	505/58	8	Cib
	A9	9	CDC → Cib
	A10	10	CDC → Cib
	BD92-00426	12	Cib
			
S. flexneri	CIP 82.48^T^	2a	CIP
	9950^*c*^	1a	SSI
	9722^*c*^	1b	SSI
	12698^*c*^	2b	SSI
	Z^*c*^	3a	SSI
	9989^*c*^	3a	SSI
	BD10-00109	3b	Cib
	8296^*c*^	4a	SSI
	9726^*c*^	4b	SSI
	8523^*c*^	5a	SSI
	8524^*c*^	5b	SSI
	9729^*c*^	6	SSI
	9951^*c*^	Y	SSI
			
S. boydii	CIP 82.50^T^	2	CIP
	9327^*c*^	1	SSI
	9850^*c*^	3	SSI
	9770^*c*^	4	SSI
	9733^*c*^	5	SSI
	9771^*c*^	6	SSI
	9734^*c*^	7	SSI
	9328^*c*^	8	SSI
	9355^*c*^	9	SSI
	9357^*c*^	10	SSI
	9359^*c*^	11	SSI
	9772^*c*^	12	SSI
	8592^*c*^	14	SSI
	10024^*c*^	15	SSI
S. sonnei	CIP 82.49^T^	ND	CIP
	9774^*c*^	Phase I	SSI
	BD13-00218	Phase I & II	Cib
	8219^*c*^	Phase II	SSI
Provisional Shigella	BD09-00375	O159	Cib
E. coli (EIEC)	CCUG 11335	O28	CCUG
	T72351^*c*^	O28	SSI
	W71750^*c*^	O28	SSI
	BD12-00018	O29	Cib
	F54157^*c*^	O64	SSI
	F54197^*c*^	O64	SSI
	BD11-00138	O102	Cib
	DSM 9027	O112ac	DSMZ
	BD11-00028	O121	Cib
	F20871^*c*^	O121	SSI
	EW227	O124	CDC → Cib
	BD13-00007	O124	Cib
	b7(D2192)^*c*^	O124	SSI
	1111-55	O136	CDC → Cib
	No2 VIR (fr1292)^*c*^	O143	SSI
	N02135 AVIR (fr1294)^*c*^	O143	SSI
	DSM 9028	O143	DSMZ
	M26020^*c*^	O144	SSI
	1624-56	O144	CDC → Cib
	BD09-00443	O152	Cib
	1184-68	O152	CDC → Cib
	BD13-00213	O159	Cib
	BD09-00375	O159	Cib
	145/46	O164	CDC → Cib
	BH 2232-5^*c*^	O172	SSI
	L119-10B	O173	SSI → Cib
	T20103^*c*^	O173	SSI
	H57237^*c*^	O+	SSI
	H19610^*c*^	O+	SSI
	BD13-00037	O untypeable	Cib
E. coli (noninvasive)	DSM 9026	O29	DSMZ
	Coli-Pecs	O135	CDC → Cib
	E10702	O167	CDC → Cib

a Shigella serotype in case of Shigella spp. or E. coli O type in case of E. coli or provisional Shigella. ND, not determined.

b CIP, Collection de l'Institut Pasteur, Paris, France; CDC, Centers for Disease Control and Prevention, Atlanta, GA, USA; Cib, Centre for Infectious Disease Control, Bilthoven, The Netherlands; CDC/SSI → Cib, historical isolates donated to Cib by the CDC or SSI, respectively, for antiserum preparation and validation from 1950s to 1980s; SSI, Statens Serum Institut, Copenhagen, Denmark; CCUG, Culture Collection, University of Göteborg, Sweden; DSMZ, Leibniz-Institut DSMZ-Deutsche Sammlung von Mikroorganismen und Zellkulturen GmbH, Braunschweig, Germany.

c Provided by F. Scheutz, SSI.

### Culture-dependent algorithm.

The culture-dependent algorithm was designed to facilitate identification and serotyping of Shigella spp. or EIEC from pure cultures up to the serotype level. It was based on the positivity of the *ipaH* gene and then subsequent profiling of earlier described phenotypical and serological features.

The isolates were cultured overnight at 37°C on Columbia sheep blood agar (CSA; bioTRADING, Mijdrecht, The Netherlands). Lysates were prepared by boiling strains in TE buffer (10 mM Tris-1 mM EDTA [pH 8.0]; Sigma-Aldrich, Zwijndrecht, The Netherlands) for 30 min. A PCR to detect the *ipaH* gene was performed using a Biometra TProfessional standard gradient thermocycler (Westburg, Leusden, The Netherlands), with the following program: 95°C for 3 min, followed by 35 cycles consisting of 95°C for 1 min, 57°C for 1 min, and 72°C for 1 min, and an elongation for 7 min at 72°C. As a mastermix, illustra PuReTaq Ready-To-Go PCR beads (GE Healthcare Life Sciences, Eindhoven, The Netherlands) were used, supplemented with the following primers designed for amplification of a conservative part of the *ipaH* gene present in all different *ipaH* alleles (20): forward primer, 5′-TGG AAA AAC TCA GTG CCT C-3′; and reverse primer, 5′-CCA GTC CGT AAA TTC ATT CTC-3′. As an internal control for presence of bacterial DNA, a conservative part of the bacterial 16S rRNA gene was amplified with the following primers: forward primer, 5′-AGA GTT TGA TCM TGG YTC AG-3′; and reverse primer, 5′-CTT TAC GCC CAR TRA WTC CG-3′. All primers were used in a final concentration of 0.2 pmol/μl.

The *ipaH*-positive isolates were subjected to the following phenotypic tests: oxidase, catalase, motility at 22°C and 37°C, growth on MacConkey agar and Salmonella Shigella agar (SS agar), gas from d-glucose, ornithine decarboxylase (ODC), indole, esculin hydrolysis, *ortho*-nitrophenyl-β-galactoside (ONPG), and fermentation of d-glucose, lactose, d-sucrose, d-xylose, d-mannitol, dulcitol, salicin, d-raffinose, and d-glycerol in Andrade peptone water (21), lysine decarboxylase (LDC [22]), and arginine dihydrolase (ADH [23]).

Next to the phenotypical tests, classical Shigella serotyping was performed with all available Shigella antisera obtained from Denka Seiken Co., Ltd. (Tokyo, Japan), complemented with S. flexneri MASF IV-1, MASF IV-2, MASF 1c, and MASF B from Reagensia AB (Solna, Sweden). If slide agglutination was negative for all polyvalent antisera or an inconclusive serotype was obtained, a suspension of the isolate was boiled for 1 h, after which slide agglutination was performed again.

Classical E. coli O serotyping was manually performed with antisera for E. coli O1 until O187, prepared as previously described (24, 25) or purchased from Statens Serum Institut (Copenhagen, Denmark). O-antigen suspensions were prepared by boiling an overnight broth culture for 1 h to inactivate the K antigen. These prepared antigens, diluted (optical density at 600 nm [OD_600_], 0.44) with formalinized (0.5%) phosphate-buffered saline (PBS), were stained with gentian violet (0.005%) and tested against the 187 O antisera in microtiter plate agglutination tests. After overnight incubation at 37°C, plates were examined against a light background, and positive reactions were titrated. O-type reactions with titers of ≥2,500, and reactions with titers until two steps lower than the reaction of the homologous standard were considered positive.

**FIG 1:**
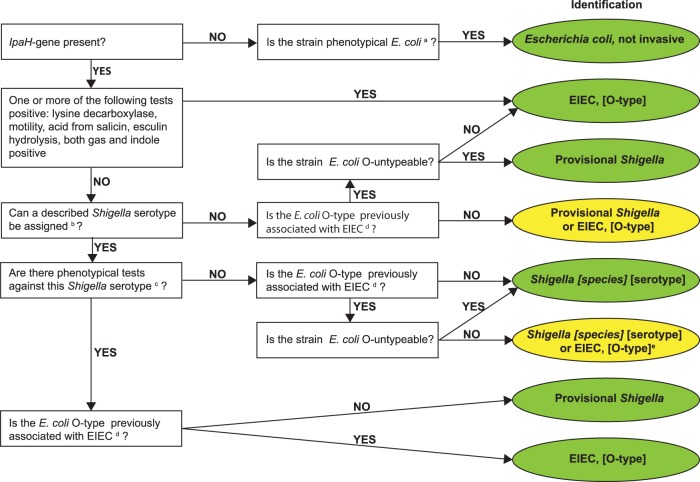
Culture-dependent algorithm. Green, definitive identification; yellow, inconclusive identification; a, Strockbine et al. (32); b, manufacturer's protocol for Shigella antisera set 1, as per Denka Seiken, Sun et al. (41, 43), and Carlin et al. (44); c, Bopp et al. (13); d, O28ac, O29, O42, O96, O112ac, O115, O121, O124, O135, O136, O143, O144, O152, O159, O164, O167, O173, and O untypeable; e, if Shigella serotype has a known relation to E. coli O type, identification is Shigella [species] [serotype]; see Ewing (31), Cheasty and Rowe (45), Liu et al. (39), and Perepelov et al. (42).

**FIG 2:**
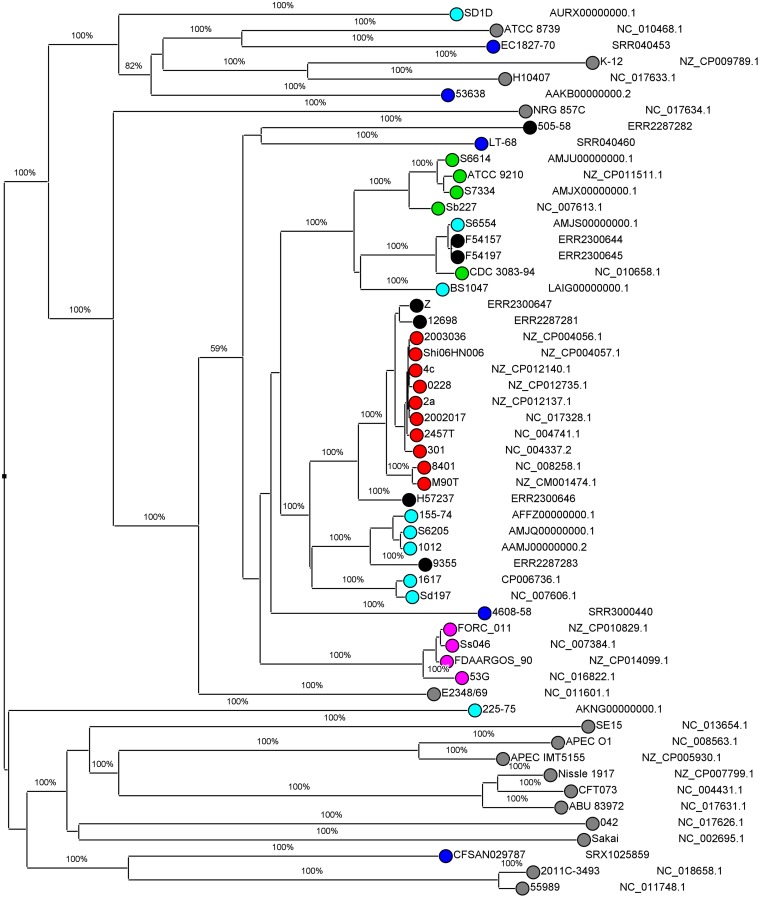
Neighbor-joining tree for core-genome MLST, with distance based on E. coli cgMLST (EnteroBase) scheme, 2,513 columns, 200× bootstrapped. Accession numbers are from GenBank or EMBL; Black, isolates 505/58, 12698, Z, 9355, F54157, F54197, and H57237; light blue, S. dysenteriae; red, S. flexneri; green, S. boydii; pink, S. sonnei; blue, EIEC; gray, other pathotypes of E. coli.

With the results of the above-described molecular, biochemical, and serological tests, an identification algorithm was applied as shown in Fig. 1, based on a previously described key (Fig. 2 in reference 26). A result was considered inconclusive if a distinction between a Shigella species and EIEC could not be made and the serotypes are not described as related.

### Molecular algorithm.

The molecular algorithm was designed to screen fecal samples for the presence of Shigella spp./EIEC quickly and accurately. However, in this study, only pure cultures were examined; thus, only the molecular part of the algorithm that follows bacterial isolation was applied (de Boer et al., unpublished data).

Briefly, lysates were prepared as described above. A real-time PCR to target the *ipaH* and the *wzx* genes of S. sonnei phase I, S. flexneri serotype 1-5, S. flexneri 6, and S. dysenteriae serotype 1 was performed on a ABI 7500 sequence detection system (Applied Biosystems, Nieuwerkerk aan den IJssel, The Netherlands), as described previously (11). Each 25-μl reaction mixture consisted of 5 μl template DNA, 1× Fast Advanced TaqMan Universal PCR master mix (Applied Biosystems), and 2.5 μg bovine serum albumin (Roche Diagnostics Netherlands B.V., Almere, The Netherlands). The primers and probes used for detection were designed based on the sequence of *wzx* genes, as described previously (27, 28). Reactions were performed under the following conditions: 50°C for 2 min, 95°C for 20 s, followed by 40 cycles of 95°C for 3 s, and 60°C for 32 s. With the result of the *ipaH* gene PCR, a distinction between Shigella/EIEC and noninvasive E. coli was made. Positivity of a *wzx* gene, in an expected ratio with a threshold cycle (*C_T_*) value of the *ipaH* gene according to copy number (20), leads to the corresponding serotype. If the *ipaH* gene had a *C_T_* value below 35 but all tested *wzx* genes were negative, the identification is inconclusive and was interpreted as EIEC, S. boydii, S. sonnei phase II, or S. dysenteriae serotype 2-15.

### Discrepancy analysis using whole-genome sequencing.

WGS analysis was performed on seven isolates to solve discrepancies between the here-proposed algorithms and original identification (Tables 2 and 3). Isolates were cultured overnight at 37°C on CSA. For each isolate, an equivalent to 5 μl of colonies was suspended in 300 μl MicroBead solution, and DNA was extracted with the UltraClean microbial DNA isolation kit (Mo Bio Laboratories, Carlsbad, CA, USA). The DNA library was prepared with the Nextera XT version 2 index kit (Illumina, San Diego, CA, USA). Subsequently, the library was sequenced on a MiSeq sequencer (Illumina, Inc.), using a MiSeq reagent kit version 3 generating 300-bp paired-end reads.

**TABLE 2:** Results of identification with culture-dependent and molecular algorithm compared to original identification^*a*^

Original identification (*n*)	Culture-dependent algorithm						Molecular algorithm					
	Concordant		Inconclusive		Discordant		Concordant		Inconclusive		Discordant	
	*n*	%	*n*	%	*n*	%	*n*	%	*n*	%	*n*	%
S. dysenteriae (12)	11 (12)	92 (100)	0	0	1 (0)	8 (0)	2	17	10	83	0	0
S. flexneri (13)	8	62	3	23	2	15	13	100	0	0	0	0
S. boydii (14)	13	93	0	0	1	7	0	0	14	100	0	0
S. sonnei (4)	4	100	0	0	0	0	2	50	2	50	0	0
EIEC (30)	26 (27)	87 (90)	1	3	3 (2)	10 (7)	0 (1)	0 (3)	29	97	1 (0)	3 (0)
E. coli, noninvasive (3)	3	100	0	0	0	0	3	100	0	0	0	0

a Concordant or discordant refers to comparison with the original identification (Table 1). For inconclusive identification, the original identification is in concordance with one of the results. Values in parentheses are the results after discrepancy analysis.

**TABLE 3:** Discrepancy analysis of isolates with discordant results based on the culture dependent algorithm

Isolate	Original identification	Results of culture-dependent algorithm and motivation	Results of molecular algorithm	Predicted E. coli serotype^*a*^	Predicted Shigella O type	Presence/absence of genes for deviant results^*b*^
505/58	S. dysenteriae serotype 8	EIEC, O untypeable; serologically S. dysenteriae 8, negative indole production against	S. dysenteriae non-O1/S. boydii/S. sonnei phase II/EIEC	O38: H26	S. dysenteriae serotype 8	*tnaCAB* cluster absent
12698	S. flexneri serotype 2b	EIEC, O untypeable; serological S. flexneri 2b, positive d-mannitol fermentation against	S. flexneri	O13:H14	S. flexneri 2b^*c*^	*mtlA*, *mtlD*, and *mtlR* genes (mannitol operon) present
Z	S. flexneri serotype 3a	EIEC, O135; S. flexneri polyvalent positive, no conclusive serotype; antigenic formula B;6	S. flexneri	O13/O135:H14	S. flexneri 1c^*d*^	NA
9355	S. boydii serotype 9	Provisional *Shigella*; serologically S. boydii 9, negative indole production against	S. dysenteriae non-O1/S. boydii/S. sonnei phase II/EIEC	No O-type genes, H14	S. boydii serotype 9	*tnaCAB* cluster present, *tnaC* and *rrlB* genes contain features essential for induction of *tnaCAB* cluster
F54157	EIEC, O64	S. sonnei, phase II; serologically and biochemically fit by repeat	S. dysenteriae non-O1/S. boydii/S. sonnei phase II/EIEC	O149:H45	S. boydii, serotype 1; no S. sonnei, S. flexneri, or S. dysenteriae O-antigen genes present	NA
F54197	EIEC, O64	S. sonnei, phase II; serologically and biochemically fit by repeat	S. dysenteriae non-O1/S. boydii/S. sonnei phase II/EIEC	O149:H45	S. boydii, serotype 1; no S. sonnei, S. flexneri or S. dysenteriae O-antigen genes present	NA
H57237	EIEC, O+	S. flexneri, serotype Yv	S. flexneri	O13:H14	S. flexneri Yv^*e*^	NA

a Using SerotypeFinder, Center for Genomic Epidemiology.

b NA, not applicable.

c *wzx_1-5_*, *gtrII*, and *gtrX* present.

d *wzx_1-5_*, *gtrI*, *gtrIc*, and *oac* present.

e *wzx_1-5_* and *lpt-O* present.

Quality control, quality trimming, and *de novo* assembly was performed using CLC Genomics Workbench, version 9.1.1 (Qiagen, Aarhus, Denmark). A quality limit of 0.01 was used in trimming, and a word size of 29 and a minimum contig length of 1,000 bp were used in *de novo* assembly. Other parameters were set as default.

E. coli O types were predicted using SerotypeFinder (Center for Genomic Epidemiology, Lyngby, Denmark). To predict the serotype of Shigella, trimmed reads of the isolates were mapped against references of the S. flexneri O-antigen genes (29) and the O-antigen gene clusters of S. dysenteriae, S. boydii, and S. sonnei (28). To our knowledge, S. dysenteriae serotypes 14 and 15 are rare, and the sequence of their O antigens is not known; therefore, these serotypes were not evaluated *in silico*. The *tnaCAB* gene cluster and *rrlB* gene were used as references for indole production from tryptophan and the *mtlA*, *mtlD,* and *mtlR* genes as references for the fermentation of d-mannitol. All genes and gene clusters were retrieved from NCBI (see Table S2 in the supplemental material). If reads mapped with one or more mutations, the functionality of the encoded proteins was assessed using ExPASy (Swiss Institute of Bioinformatics [SIB] [30]) and BLASTp (NCBI, Bethesda, MD, USA).

The *de novo* assemblies were imported in SeqSphere^+^ version 3.5.1 (Ridom GmbH, Münster, Germany), including reference genomes retrieved from NCBI, to assess the homologies of the discrepant strains with the references. A comparison of the sequences was made using the E. coli core-genome multilocus sequence typing (cgMLST) genotyping scheme, which is based on the EnteroBase Escherichia/Shigella cgMLST version 1 scheme (https://enterobase.warwick.ac.uk/species/index/ecoli). The resulting comparison table was imported in BioNumerics, version 7.6.3 (Applied Maths, NV), and a neighbor joining tree was inferred using 200× bootstrap resampling. The tree with the highest resampling support was calculated. The accession numbers of all sequences are depicted in Fig. 2.

### Accession number(s).

The sequences of discrepant isolates were submitted to the European Nucleotide Archive (ENA, EMBL-EBI, Cambridge, United Kingdom) as study no. PRJEB24877 with accession numbers ERR2287281 (isolate 12698), ERR2287282 (isolate 505/58), ERR2287283 (isolate 9355), ERR2300644 (isolate F54157), ERR2300645 (isolate F54197), ERR2300646 (isolate H57237), and ERR2300647 (isolate Z) (https://www.ebi.ac.uk/ena).

## RESULTS

### Culture-dependent algorithm.

With the culture-dependent algorithm, an inconclusive result was obtained for four isolates (Table 2). For these isolates, a distinction between EIEC and either S. flexneri, S. boydii, or S. dysenteriae was impossible, and the Shigella O type has no known relationship to the E. coli O type. Only S. sonnei and noninvasive E. coli isolates were completely concordant with the original identification, including the inconclusive results. The obtained percentages of concordance were 92%, 85%, 93%, and 90% for S. dysenteriae, S. flexneri, S. boydii, and EIEC isolates, respectively (Table 2).

### Molecular algorithm.

For 55 isolates (72%), only the *ipaH* gene was detected and none of the assessed *wzx* genes detected using the molecular algorithm. These isolates were binned in the rest group, meaning they can be either EIEC, S. sonnei phase II, S. boydii, or S. dysenteriae serotypes other than 1. All isolates except for one EIEC strain (97%) were identified in concordance with the original identification or had an inconclusive result, of which one of the results was in concordance with original identification (Table 2). One isolate had a discordant identification, although the result of the molecular algorithm was in concordance with the culture-dependent algorithm (strain H57237, Table 3).

### Discrepancy analysis of discordant results.

Seven isolates showed discordant results with the original identification using the culture-dependent algorithm (Table 2), and a discrepancy analysis using WGS was carried out (Table 3). The predicted E. coli and Shigella serotypes and the presence of genes that encode for specific features are displayed in Table 3, as well as the results of the two tested algorithms (Table 3). The clustering of the discrepant isolates with reference isolates is shown in the cgMLST analysis (Fig. 2).

In the discrepancy analysis of isolate 505/58, WGS data confirmed the serotype as determined at original identification and with the culture-dependent algorithm, as the predicted serotypes are E. coli O38 and S. dysenteriae serotype 8, which are related to each other (31). However, because indole was negative, while all strains described in the literature from S. dysenteriae serotype 8 are capable of producing indole (13, 31), and because the E. coli O antigen is not typeable phenotypically (32), isolate 505/58 was identified as EIEC O-untypeable using the culture-dependent algorithm. WGS data confirmed that the *tnaCAB* cluster, which contains the functional genes for the production of indole from tryptophan (33), is absent in 505/58. cgMLST showed that isolate 505/58 clustered with an EIEC reference genome and not with other S. dysenteriae reference genomes in this analysis (Fig. 2). The molecular algorithm placed 505/58 in the rest group, which is in concordance with the original identification, as well as with the culture-dependent algorithm (Table 3). The clustering combined with the absence of the *tnaCAB* cluster indicates that 505/58 was originally misidentified as S. dysenteriae or that it has lost the *tnaCAB* cluster over time.

With isolate 12698, WGS data confirmed the serotype as determined at the original identification and with the culture-dependent algorithm to be S. flexneri serotype 2b. The molecular algorithm confirmed these results, as it detected the presence of the *wzx_1-5_* gene (Table 3). However, using the culture-dependent algorithm, 12698 was repeatedly d-mannitol positive, while all described S. flexneri serotype 2b isolates are d-mannitol negative (13, 31). Because d-mannitol was positive and the E. coli O type is untypeable (32), 12698 was identified as EIEC O untypeable using the culture-dependent algorithm. The WGS data confirmed the d-mannitol-positive result, as it detected the *mtlA* and *mtlD* genes and its regulator *mtlR* (34). However, despite the positive result of d-mannitol fermentation, isolate 12698 clustered with S. flexneri reference isolates using cgMLST (Fig. 2), supporting the original identification, as well as the classical and *in silico* serotyping to designate isolate 12698 S. flexneri serotype 2b.

Discrepancy analysis using WGS for isolate Z added an additional identification instead of confirming one of the other results. Isolate Z was originally identified as S. flexneri 3a, while with the culture-dependent algorithm, the isolate fit phenotypically to S. flexneri 3a but had a serologically inconclusive serotype with antigenic formula B;6. Because the Shigella antigenic formula was inconclusive and the E. coli O type was O135 (14), isolate Z was identified as EIEC O135 with the culture-dependent algorithm. WGS analysis detected the presence of the following S. flexneri genes and clusters in isolate Z: *wzx_1-5_*, *oac*, *gtrI,* and *gtr1C*, resulting in S. flexneri serotype 1c (Table 3). Although the completely conserved *gtrI* and *gtrIc* clusters are present, including the *gtrA* and *gtrB* genes (35, 36), with classical Shigella serotyping, agglutination with type I and MASF 1c antisera was absent. In the cgMLST analysis, isolate Z clustered with S. flexneri reference isolates (Fig. 2). The molecular algorithm identified isolate Z as *S. flexneri*; however, this algorithm is not able to distinguish different serotypes (Table 3). To summarize, classical and *in silico* serotyping, cgMLST analysis, and the result of the molecular algorithm confirmed the original identification of isolate Z as S. flexneri but with discordances in its serotype.

In the discrepancy analysis of isolate 9355, WGS data confirmed the serotype as determined at the original identification and with the culture-dependent algorithm to be S. boydii serotype 9. However, because indole is negative, while this should be positive for S. boydii serotype 9 (13, 31), and the E. coli O type is O132, which has never been associated with EIEC, isolate 9355 was provisionally identified as Shigella using the culture-dependent algorithm. The molecular algorithm placed 9355 in the rest group, which is in concordance with original identification as well as with the culture-dependent algorithm (Table 3). The WGS data suggest that the whole *tnaCAB* cluster is present in isolate 9355 and contains the indole production genes *tnaA*, *tnaB*, and *tnaC* (33), which all encode functional proteins. Furthermore, all necessary features for the induction of *tnaA* and *tnaB* genes are present in the *tnaC* and *rrlB* genes (37, 38). The mechanism that hinders the production of indole could not be determined by assessing the presence or absence of functional genes and features and is a subject for further investigation. Isolate 9355 clustered with S. dysenteriae genomes in the cgMLST analysis. As clustering of S. boydii with S. dysenteriae was described before (5), cgMLST supports the original identification and the classical and *in silico* serotype to designate isolate 9355 S. boydii serotype 9.

For isolates F54157 and F54197, discrepancy analysis using WGS added an additional identification instead of confirming one of the other results. They were originally identified as EIEC O64 and as S. sonnei phase II in the culture-dependent algorithm; however, they were predicted to be E. coli O149 and S. boydii serotype 1 with WGS data (Table 3), which were described as identical antigens (31, 39). Agglutination with S. sonnei phase II antiserum in the culture-dependent algorithm could be explained by linkage between enterobacterial common antigen, which is a surface antigen present in Enterobacteriaceae, and S. sonnei phase II core oligosaccharide (40). With the molecular algorithm, isolates F54157 and F54197 were binned in the rest group, which is in concordance with the original identification, with the culture-dependent algorithm and with WGS data. Evaluation of the S. boydii serotype 1 O-antigen cluster in the WGS data in more detail showed intact *wzx* and *wzy* genes but major deletions in the *rmlB* gene for both isolates, explaining the lack of expression of the S. boydii serotype 1/E. coli O149 phenotype (39). In the cgMLST analysis, strains F54157 and F54197 clustered with S. dysenteriae and S. boydii strains. Overall, the discrepancy analysis based on WGS showed that isolates F54157 and F54197 were originally misidentified as EIEC with O type O64 and misidentified with the culture-dependent algorithm as S. sonnei phase II.

Isolate H57237 was originally identified as EIEC; however, both algorithms used in this study identified this isolate as S. flexneri. The serotype of H57237 is Yv, as determined by the culture-dependent algorithm and confirmed by the WGS analysis (Table 3). Serotype Yv has only recently been described (41), and probably, the original identification of this isolate predates the discovery of this novel serotype.

The discrepancy analysis showed that isolates H57237, F54157, F54197, and 505/58 might be misidentified during the original identification (Table 3 and Fig. 2). The results of the comparison of the molecular and culture-dependent algorithms with the original identification were corrected for these findings and are displayed in parentheses in Table 2.

## DISCUSSION

After discrepancy analysis, the identification of S. dysenteriae, S. sonnei, and noninvasive E. coli isolates with the culture-dependent algorithm was 100% in concordance with the original identification, including the inconclusive results. For S. flexneri, S. boydii, and EIEC isolates, the concordance was 85%, 93%, and 93%, respectively.

With the molecular algorithm, 100% of the isolates were identified in concordance with the original identification after discrepancy analysis (Table 3). However, its resolution for certain serotypes is low, as it does not allow specific detection of EIEC, S. boydii, S. sonnei phase II, and S. dysenteriae serotype 2-15. Another limitation is that cross-reactivity of Shigella and E. coli O antigens is described. The primers from the *S. dysenteriae wzx* gene are likely to amplify the E. coli O-antigen clusters O1, O120, and O148 (31, 39), and the primers from the *S. flexneri wzx_1-5_* gene will probably amplify the E. coli O-antigen clusters O1, O13, O16, O19, O62, O69, O73, O135, and O147 (31, 42). Of all these E. coli O types, only O135 is described as an EIEC-associated O type; none of the other E. coli O types are likely to possess the *ipaH* gene and are therefore not considered to be Shigella spp. or EIEC in the molecular algorithm. Nevertheless, EIEC with O type O135 cannot be separated from S. flexneri. However, this is overcome in a diagnostic setting by targeted culture from the fecal samples prompted by the results of the molecular part of the algorithm. If an isolate is selected, it is identified based on a few phenotypical key features and agglutination with Shigella and EIEC polyvalent antisera. If no isolate is selected, the physician will receive a report that Shigella spp. or EIEC is detected but without specifications about species or serotype.

One of the strengths of this study is the discrepancy analysis with WGS. This analysis is able to confirm one of the determined identities of isolates 505/58, 12698, 9355, and H57237. In contrast to those isolates, for isolates Z, F54157, and F54197, the discrepancy analysis with WGS added an extra identification result, therefore complicating the identification further instead of clarifying it.

Isolates 12698, 9355, and 505/58 were serological congruent using all identification methods, including WGS, but had one phenotypical test in discordance with their serotype (Table 3), resulting in a different identification by the culture-dependent algorithm. Phenotypical properties of a serotype are described by testing multiple isolates of the same serotype. There is not necessarily a causal connection between the serotype and the results of phenotypic tests, and phenotypic variability increases with the number of tested isolates. If the culture-dependent algorithm was applied less stringently and one phenotypical test against it was allowed, the above-described isolates were correctly identified. However, disregarding phenotypic test results should be considered carefully, because some phenotypic traits are set as defining for genus or species, for instance, the absence of LDC or d-mannitol fermentation, which are genus specific for Shigella or set as species specific for *S. dysenteriae,* respectively. The results of these species specific phenotypic tests should not be disregarded.

A limitation of this study is that only a few isolates of every species were used, and it is desirable to test more isolates with the proposed algorithms in the future. However, rare serotypes were difficult to obtain, and one can debate to omit these rare serotypes for test evaluation, because they are not frequently encountered in clinical diagnostics.

The here-described culture-dependent algorithm outperforms the previously described method based on the detection of the *uidA* gene and the *lacY* gene (16) that only correctly identified *in silico* 100% of S. sonnei, 92% of S. flexneri, 86% of S. boydii, 80% of S. dysenteriae, 77% of noninvasive E. coli, and 62% of EIEC isolates (6). In addition, the *lacY* gene approach is able to distinguish organisms to the genus level (16); therefore, its resolution is lower than that of the culture-dependent algorithm described in this study.

The previously described k-mer-based method outperforms the here-described culture-dependent algorithm for the identification of Shigella species, because it identified 100% of all Shigella species isolates in concordance with biochemical and serological profiling. In contrast, for identification of EIEC isolates, the proposed culture-dependent algorithm is superior, identifying 93% of EIEC isolates according to original identification, against 81.5% of EIEC isolates with the k-mer based approach (18). Furthermore, for the k-mer-based method, sequencing of whole genomes and subsequent bioinformatics analysis are required, making it less applicable in low-resource settings, where Shigella spp. are encountered frequently. Moreover, to match the resolution of the culture-dependent algorithm, extra analyses should be added to the k-mer-based method in order to determine the *in silico* serotype.

This study shows again that species differentiation of Shigella spp. and E. coli is challenging, as other studies have concluded before (5, 6, 18). With some isolates, differentiation is impossible, as evidenced by the percentage of isolates (5%) for which identification is inconclusive with the culture-dependent algorithm. Using the molecular algorithm, 71% of the isolates resulted in an inconclusive identification; however, this algorithm was not designed for use in the distinction between EIEC, S. boydii, S. sonnei phase II, and S. dysenteriae serotype 2-15. Nevertheless, the molecular algorithm would be sufficient for use in a developed country, because a recent study in The Netherlands (R. F. de Boer, unpublished data) showed that in 80% of *ipaH* gene-positive fecal samples, S. sonnei or S. flexneri is present. For use in other regions, the concept of the molecular algorithm can be adjusted to their particular needs; targets of *wzx* genes of S. dysenteriae and S. boydii can be added or the whole procedure can be redefined to a conventional PCR platform if real-time platforms are unavailable.

In conclusion, although not perfect, the proposed algorithms are capable of identifying most Shigella sp. and EIEC isolates. The molecular algorithm is fast and accurate and is suitable for daily application in diagnostic laboratories, as it can be performed with standard PCR equipment; however, its resolution for certain serotypes is low. The culture-dependent algorithm is more time-consuming, and many phenotypical tests and antisera are required, yet the resolution is high for all serotypes. If a desirable complete identification cannot be obtained with the molecular algorithm, the culture-dependent algorithm can be applied by a reference laboratory to obtain a higher resolution.

Despite the genetic relationship of Shigella spp. and EIEC, causing difficulties for identification, differentiation is still necessary for epidemiological and surveillance purposes because of current guidelines for infectious disease control. One can speculate if guidelines need to be adjusted, but evidence for guideline optimization with regard to infections with EIEC is currently lacking. In the future, the impact of infections with EIEC on individual patients and on public health should be further investigated to assess if it is justified that surveillance measures and control guidelines for infections with EIEC are different from those of shigellosis.

## Supplementary Material

Supplemental file 1
