# Supplementary material for: Evaluation of a Culture-Dependent Algorithm and a Molecular Algorithm for Identification of Shigella spp., Escherichia coli, and Enteroinvasive E. coli
Source: J Clin Microbiol. 2018 Sep 25;56(10):e00510-18. doi: 10.1128/JCM.00510-18 (PMC6156305; doi:10.1128/JCM.00510-18)
Supplement: Supplemental file 1 [file zjm999096106s1.pdf]

Supplementary table 1. Reference sequences

**Description**	**Accession number**	**Reference(s)**
E. coli tnaCAB cluster	NC_000913	(1, 2)
E. coli rrlB gene (23S rRNA gene)	NC_000913	(1, 3)
S. flexneri mtlA, mtlD and *mtlR* genes	NC_004741	(4, 5)
S. dysenteriae type 1 O-antigen cluster	L07293	(6)
S. dysenteriae type 2 O-antigen cluster	EU296404	(7)
S. dysenteriae type 3 O-antigen cluster	EU296415	(7)
S. dysenteriae type 4 O-antigen cluster	EU296402	(7)
S. dysenteriae type 5 O-antigen cluster	EU294174	(7)
S. dysenteriae type 6 O-antigen cluster	EU296414	(7)
S. dysenteriae type 7 O-antigen cluster	AY380835	(8)
S. dysenteriae type 8 O-antigen cluster	EU294166	(7)
S. dysenteriae type 9 O-antigen cluster	EU296416	(7)
S. dysenteriae type 10 O-antigen cluster	EU294178	(7)
S. dysenteriae type 11 O-antigen cluster	EU294172	(7)
S. dysenteriae type 12 O-antigen cluster	EU294169	(7)
S. dysenteriae type 13 O-antigen cluster	EU294167	(7)
S. flexneri lptO gene	NC_017320	(9)
S. flexneri oac gene	AF547987	(10)
S. flexneri gtrI gene cluster	AF139596	(11)
S. flexneri gtrIV gene cluster	AF288197	(12)
S. flexneri gtrII gene cluster	AF021347	(13)
S. flexneri gtrV gene cluster	U82619	(14)
S. flexneri $wzx_{1\text{-}5}$ gene (*rfbE*)	AE005674	(15)
S. flexneri gtrX gene	L05001	(16)
S. flexneri gtrIc gene cluster	FJ905303	(17)
S. flexneri 6 O antigen gene cluster	EU294165	(7)
S. boydii type 1 O antigen cluster	AY630255	(18)
S. boydii type 2 O antigen cluster	EU296418	(7)
S. boydii type 3 O antigen cluster	EU296407	(7)
S. boydii type 4 O antigen cluster	AF402312	(19)
S. boydii type 5 O antigen cluster	AF402313	(19)
S. boydii type 6 O antigen cluster	AF402314	(19)
S. boydii type 7 O antigen cluster	EU296411	(7)
S. boydii type 8 O antigen cluster	EU294163	(7)
S. boydii type 9 O antigen cluster	AF402315	(19)
S. boydii type 10 O antigen cluster	AY693427	(20)
S. boydii type 11 O antigen cluster	AY529126	(21)
S. boydii type 12 O antigen cluster	EU296406	(7)
S. boydii type 13 O antigen cluster	AY369140	(22)
S. boydii type 14 O antigen cluster	EU296409	(7)
S. boydii type 15 O antigen cluster	EU296412	(7)
S. boydii type 16 O antigen cluster	DQ371800	(23)
S. boydii type 17 O antigen cluster	DQ875941	(24)
S. boydii type 18 O antigen cluster	AY948196	(25)

References supplementary Table 1

1. Riley M, Abe T, Arnaud MB, Berlyn MK, Blattner FR, Chaudhuri RR, Glasner JD, Horiuchi T, Keseler IM, Kosuge T, Mori H, Perna NT, Plunkett G, 3rd, Rudd KE, Serres MH, Thomas GH, Thomson NR, Wishart D, Wanner BL. 2006. Escherichia coli K-12: a cooperatively developed annotation snapshot--2005. Nucleic Acids Res 34:1-9.
2. Li G, Young KD. 2015. A new suite of tnaA mutants suggests that Escherichia coli tryptophanase is regulated by intracellular sequestration and by occlusion of its active site. BMC Microbiol 15:14.
3. Cruz-Vera LR, Rajagopal S, Squires C, Yanofsky C. 2005. Features of ribosome-peptidyl-tRNA interactions essential for tryptophan induction of tna operon expression. Mol Cell 19:333-43.
4. Wei J, Goldberg MB, Burland V, Venkatesan MM, Deng W, Fournier G, Mayhew GF, Plunkett G, 3rd, Rose DJ, Darling A, Mau B, Perna NT, Payne SM, Runyen-Janecky LJ, Zhou S, Schwartz DC, Blattner FR. 2003. Complete genome sequence and comparative genomics of Shigella flexneri serotype 2a strain 2457T. Infect Immun 71:2775-86.
5. Tan K, Clancy S, Borovilos M, Zhou M, Horer S, Moy S, Volkart LL, Sassoon J, Baumann U, Joachimiak A. 2009. The mannitol operon repressor MtlR belongs to a new class of transcription regulators in bacteria. J Biol Chem 284:36670-9.

6. Sturm S, Jann B, Jann K, Fortnagel P, Timmis KN. 1986. Genetic and biochemical analysis of Shigella dysenteriae 1 O antigen polysaccharide biosynthesis in Escherichia coli K-12: structure and functions of the rfb gene cluster. Microb Pathog 1:307-24.
7. Liu B, Knirel YA, Feng L, Perepelov AV, Senchenkova SN, Wang Q, Reeves PR, Wang L. 2008. Structure and genetics of Shigella O antigens. FEMS Microbiol Rev 32:627-53.
8. Feng L, Tao J, Guo H, Xu J, Li Y, Rezwan F, Reeves P, Wang L. 2004. Structure of the Shigella dysenteriae 7 O antigen gene cluster and identification of its antigen specific genes. Microb Pathog 36:109-15.
9. Ye C, Lan R, Xia S, Zhang J, Sun Q, Zhang S, Jing H, Wang L, Li Z, Zhou Z, Zhao A, Cui Z, Cao J, Jin D, Huang L, Wang Y, Luo X, Bai X, Wang Y, Wang P, Xu Q, Xu J. 2010. Emergence of a new multidrug-resistant serotype X variant in an epidemic clone of Shigella flexneri. J Clin Microbiol 48:419-26.
10. Clark CA, Beltrame J, Manning PA. 1991. The oac gene encoding a lipopolysaccharide O-antigen acetylase maps adjacent to the integrase-encoding gene on the genome of Shigella flexneri bacteriophage Sf6. Gene 107:43-52.
11. Adhikari P, Allison G, Whittle B, Verma NK. 1999. Serotype 1a O-antigen modification: molecular characterization of the genes involved and their novel organization in the Shigella flexneri chromosome. J Bacteriol 181:4711-8.
12. Adams MM, Allison GE, Verma NK. 2001. Type IV O antigen modification genes in the genome of Shigella flexneri NCTC 8296. Microbiology 147:851-60.

13. Mavris M, Manning PA, Morona R. 1997. Mechanism of bacteriophage SfII-mediated serotype conversion in Shigella flexneri. Mol Microbiol 26:939-50.

14. Huan PT, Bastin DA, Whittle BL, Lindberg AA, Verma NK. 1997. Molecular characterization of the genes involved in O-antigen modification, attachment, integration and excision in Shigella flexneri bacteriophage SfV. Gene 195:217-27.

15. Jin Q, Yuan Z, Xu J, Wang Y, Shen Y, Lu W, Wang J, Liu H, Yang J, Yang F, Zhang X, Zhang J, Yang G, Wu H, Qu D, Dong J, Sun L, Xue Y, Zhao A, Gao Y, Zhu J, Kan B, Ding K, Chen S, Cheng H, Yao Z, He B, Chen R, Ma D, Qiang B, Wen Y, Hou Y, Yu J. 2002. Genome sequence of Shigella flexneri 2a: insights into pathogenicity through comparison with genomes of Escherichia coli K12 and O157. Nucleic Acids Res 30:4432-41.

16. Verma NK, Verma DJ, Huan PT, Lindberg AA. 1993. Cloning and sequencing of the glucosyl transferase-encoding gene from converting bacteriophage X (SFX) of Shigella flexneri. Gene 129:99-101.

17. Stagg RM, Tang SS, Carlin NI, Talukder KA, Cam PD, Verma NK. 2009. A novel glucosyltransferase involved in O-antigen modification of Shigella flexneri serotype 1c. J Bacteriol 191:6612-7.

18. Tao J, Wang L, Liu D, Li Y, Bastin DA, Geng Y, Feng L. 2005. Molecular analysis of Shigella boydii O1 O-antigen gene cluster and its PCR typing. Can J Microbiol 51:387-92.

19. Wang L, Qu W, Reeves PR. 2001. Sequence analysis of four Shigella boydii O-antigen loci: implication for Escherichia coli and Shigella relationships. Infect Immun 69:6923-30.

20. Senchenkova SN, Feng L, Yang J, Shashkov AS, Cheng J, Liu D, Knirel YA, Reeves PR, Jin Q, Ye Q, Wang L. 2005. Structural and genetic characterization of the Shigella boydii type 10 and type 6 O antigens. J Bacteriol 187:2551-4.

21. Tao J, Feng L, Guo H, Li Y, Wang L. 2004. The O-antigen gene cluster of Shigella boydii O11 and functional identification of its wzy gene. FEMS Microbiol Lett 234:125-32.

22. Feng L, Senchenkova SN, Yang J, Shashkov AS, Tao J, Guo H, Zhao G, Knirel YA, Reeves P, Wang L. 2004. Structural and genetic characterization of the Shigella boydii type 13 O antigen. J Bacteriol 186:383-92.

23. Liu B, Senchenkova SN, Feng L, Perepelov AV, Xu T, Shevelev SD, Zhu Y, Shashkov AS, Zou M, Knirel YA, Wang L. 2006. Structural and molecular characterization of Shigella boydii type 16 O antigen. Gene 380:46-53.

24. Senchenkova SN, Feng L, Wang Q, Perepelov AV, Qin D, Shevelev SD, Ren Y, Shashkov AS, Knirel YA, Wang L. 2006. Structural and genetic characterization of Shigella boydii type 17 O antigen and confirmation of two new genes involved in the synthesis of glucolactilic acid. Biochem Biophys Res Commun 349:289-95.

25. Feng L, Senchenkova SN, Wang W, Shashkov AS, Liu B, Shevelev SD, Liu D, Knirel YA, Wang L. 2005. Structural and genetic characterization of the Shigella boydii type 18 O antigen. Gene 355:79-86.

26. Shepherd JG, Wang L, Reeves PR. 2000. Comparison of O-antigen gene clusters of Escherichia coli (Shigella) sonnei and Plesiomonas shigelloides O17: sonnei gained its current plasmid-borne O-antigen genes from P. shigelloides in a recent event. Infect Immun 68:6056-61.
